# Onboard Image Processing System for Hyperspectral Sensor

**DOI:** 10.3390/s151024926

**Published:** 2015-09-25

**Authors:** Hiroki Hihara, Kotaro Moritani, Masao Inoue, Yoshihiro Hoshi, Akira Iwasaki, Jun Takada, Hitomi Inada, Makoto Suzuki, Taeko Seki, Satoshi Ichikawa, Jun Tanii

**Affiliations:** 1 NEC Space Technologies, Ltd., 1-10, Nisshin-cho, Fuchu, Tokyo 183-8551, Japan; E-Mails: k-moritani@bk.jp.nec.com (K.M.); m-inoue@pi.jp.nec.com (M.I.); y-hoshi@uf.jp.nec.com (Y.H.); 2 Research Center for Advanced Science and Technology, the University of Tokyo, 4-6-1 Komaba, Meguro-ku, Tokyo 153-8904, Japan; E-Mail: aiwasaki@sal.rcast.u-tokyo.ac.jp; 3 Central Research Laboratory, NEC Corporation, 1753, Shimonumabe, Nakahara-Ku, Kawasaki, Kanagawa 211-8666, Japan; E-Mail: j-takada@bc.jp.nec.com; 4 Space Systems Division, NEC Corporation, 1-10, Nisshin-cho, Fuchu, Tokyo 183-8551, Japan; E-Mail: h-inada@bx.jp.nec.com; 5 Institute of Space Astronautical Science (ISAS), Japan Aerospace Exploration Agency (JAXA), 3-1-1 Yoshinodai, Chuo-ku, Sagamihara, Kanagawa 252-5210, Japan; E-Mail: suzuki.makoto@jaxa.jp; 6 Aerospace Research and Development Directorate, Japan Aerospace Exploration Agency (JAXA), 2-1-1 Sengen, Tsukuba, Ibaraki 305-8505, Japan; E-Mails: seki.taeko@jaxa.jp (T.S.); ichikawa.satoshi@jaxa.jp (S.I.); 7 Japan Space Systems, 3-5-8 Shibakoen, Minato-ku, Tokyo 105-0011, Japan; E-Mail: Tanii-Jun@jspacesystems.or.jp

**Keywords:** hyperspectral sensor, Golomb-Rice coding, hierarchical prediction, lossless image compression, predictive coding, resolution scaling, onboard correction, smile correction

## Abstract

Onboard image processing systems for a hyperspectral sensor have been developed in order to maximize image data transmission efficiency for large volume and high speed data downlink capacity. Since more than 100 channels are required for hyperspectral sensors on Earth observation satellites, fast and small-footprint lossless image compression capability is essential for reducing the size and weight of a sensor system. A fast lossless image compression algorithm has been developed, and is implemented in the onboard correction circuitry of sensitivity and linearity of Complementary Metal Oxide Semiconductor (CMOS) sensors in order to maximize the compression ratio. The employed image compression method is based on Fast, Efficient, Lossless Image compression System (FELICS), which is a hierarchical predictive coding method with resolution scaling. To improve FELICS’s performance of image decorrelation and entropy coding, we apply a two-dimensional interpolation prediction and adaptive Golomb-Rice coding. It supports progressive decompression using resolution scaling while still maintaining superior performance measured as speed and complexity. Coding efficiency and compression speed enlarge the effective capacity of signal transmission channels, which lead to reducing onboard hardware by multiplexing sensor signals into a reduced number of compression circuits. The circuitry is embedded into the data formatter of the sensor system without adding size, weight, power consumption, and fabrication cost.

## 1. Introduction

Onboard image processing is often a mandatory function of hyperspectral sensors for Earth observation satellites. Fast and compact circuitry is required for onboard compression in order to transmit hyperspectral sensor data from a satellite to ground stations due to the following factors. At first, a new dimension is added on the two-dimensional sensor image because the image data derived from the sensor include spectral information in addition to the spatial information, and the amount of data increases significantly. As a result, a hyperspectral sensor accommodates more than 100 spectral channels [1,2,3,4], and, consequently, more than several tens of times of conventional multispectral sensor data or more than 100 times of conventional panchromatic sensor data must be transmitted from a satellite to ground stations. Secondly, users of hyperspectral sensor data require no degradations on the image data quality, even though the resources of the sensor system are limited. Therefore, a lossless image data compression function should be realized without adding remarkable size, mass, and power consumption for onboard electronics of satellites. Finally, the onboard calibration system for sensor image data is required in order to keep the precision of sensor data during the image compression process as well as the high compression ratio.

Various methods have been investigated for image data compression [5]. Fast image compression capability using a powerful GPU (Graphics Processing Unit) was reported [6]; however, such implementation requires higher power consumption and larger footprints. A low complexity Field Programmable Gate Array (FPGA) implementation is preferred in order to keep small a footprint. The decorrelation of sensor data is pursued [7] in order to improve data compression efficiency, and such decorrelation characteristics should always be exploited with a camera rate processing speed for arbitrary natural images.

The algorithm of image data compression is the key issue for onboard electronics. The faster onboard electronics compress image data, the more numbers of channels can be processed by multiplexing sensor input channels. The footprint of electronic circuitry for image data compression should be as small as possible in order to realize the low heat dissipation, light weight, and small size required for large scale optical sensors.

A fast and small-footprint lossless image compression algorithm has been developed [1,2]. The new algorithm can be implemented either in software or in hardware. The compression function is implemented as hardware for our hyperspectral sensor in order to maintain the bandwidth within the capacity of downlink transmission channels through the camera rate processing speed for all sensor input channels without losing both spectral and spatial information. Fast and low-complexity characteristics enable eliminating the independent onboard data compression unit. The compression circuitry has been achieved to be embedded in the sensor signal encoding formatter, which led to a significant mass reduction.

The calibration algorithm for the radiance of each element of the sensors and for the spectral misregistration (smile characteristics) of the optical subsystem is optimized in order to realize camera rate processing with compact hardware implementation.

The compression algorithm and the calibration algorithm are compact enough to implement them into Field Programmable Gate Array (FPGA) rather than Application Specific Integration Circuit (ASIC). In consequence, the circuitry for compression, luminance calibration, and smile characteristics calibration are embedded into the front-end of the electronics unit.

## 2. Hyperspectral Imager SUIte (HISUI)

### 2.1. Instrument Overview

HISUI is a next-generation sensor in Japan that will be on board in 2017 or later. HISUI consists of a hyperspectral-radiometer unit with precise spectral resolution, a multi-spectral radiometer with precise spatial resolution, and a control unit [3,4]. The HISUI project is a heritage of the Advanced Space-borne Thermal Emission and Reflection Radiometer (ASTER) [8]. It was launched in 1999 and has been operating for well over 15 years in orbit, although the thermal bands are not included. A narrow-bandwidth multi-band imager of ASTER in the shortwave infrared region is inherited by HISUI as a hyperspectral sensor.

The hyperspectral radiometer covers the spectral range from the visible to the short-wavelength infrared region (0.4–2.5 μm). It covers the spectral range of the VNIR (visible and near infra-red: 400–970 nm) and SWIR (short wavelength infra-red: 900–2500 nm) regions by 186 bands with a 30 m ground sampling distance and a 30 km swath width. The multi-spectral radiometer scans the wide swath of 90 km with the ground sampling distance of 5 m.

The physical value of spectral sampling intervals is 2.5 nm for VNIR and 6.25 nm for SWIR, and the on-board calibration accuracy for spectral performance is less than 0.2 nm for VNIR and less than 0.625 nm for SWIR. In order to satisfy these requirements and the spectral sampling uniformity, the two sets of reflective grating-type spectrometers for VNIR and SWIR are adopted.

The flight model of VNIR and SWIR radiometers have already been developed, and the spectral and radiometric performance of these radiometers have been confirmed. The flight model contains the VNIR and SWIR spectrometers and the VNIR and SWIR detector assemblies with a mechanical cooler for SWIR. The flight models of electronics units for VNIR and SWIR are under development. These units include signal processing circuitry and an on-board calibration source. The functional evaluation model and the engineering model have already been developed prior to the manufacturing of the flight models.

### 2.2. Radiometer Overview

As for the hyperspectral sensor, the emission from the Earth is guided into the optical telescope and is focused on the slits of the two spectrometers. One is for the visible and near infrared (VNIR) radiometer, and the other is for the short wavelength infrared (SWIR) radiometer. The Silicon CMOS detector is adopted for the VNIR detector, whereas the Mercury Cadmium Telluride (MCT) (HgCdTe) is adopted for the SWIR detector. The latter is cooled with the Stirling-type cryo-cooler.

Table 1 shows the specifications of the hyperspectral radiometer. The designed signal-to-noise ratio is 450 and 300 for VNIR and SWIR, respectively. The diameter of the telescope is designed to be around 30 cm and the F-number is 2.2 in order to satisfy the high signal-to-noise ratio requirement.

**Table 1:** The specifications of HISUI.

Instrument Type	Hyperspectral Sensor		Multi-Spectral Sensor
	VNIR	SWIR	
IFOV (Spatial resolution) * Note 1	48.5 μrad (30 m)		8.1 μrad (5 m)
FOV (Swath width) * Note 1	48.5 mrad (Around 30 km)		144.7 mrad (Around 90 km)
Observation Interval/Period	≤4.36 ms		≤0.73 ms
Wavelength region and number of bands	400–970 nm ≥ 57 bands	900–2500 nm ≥ 128 bands	Band1: 485 nm Band2: 560 nm Band3: 660 nm Band4: 830 nm
Spectral resolution (sampling)	10 nm	12.5 nm	Band1: 70 nm Band2: 80 nm Band3: 60 nm Band4: 140 nm
Dynamic range	Saturated at ≥70% Albedo	Saturated at ≥70% Albedo	Saturated at ≥70% Albedo
SNR	≥450 @620 nm	≥300 @2100 nm	≥200 (for each band)
MTF	≥0.2	≥0.2	≥0.3
Smile and Keystone	≤1 image pixel	≤1 image pixel	N/A
Calibration Accuracy (Radiometric)	Absolute: ±5% Among bands: ±2%	Absolute: ±5% Among bands: ±2%	Absolute: ±5% Among bands: ±2%
Calibration Accuracy (Spectral)	0.2 nm	0.625 nm	N/A
Data rate	68.4–76.0 Mbps/ch (typ) 107.6 Mbps/ch (max)	128.4–143.5 Mbps/ch (typ) 206.8 Mbps/ch (max)	207.5 Mbps/ch (nom)
Quantization	12 bit		12 bit

* Note 1: Assumed satellite altitude is 618.2 km for spatial resolution and swath width.

### 2.3. Signal Processor

Signal processing functions of HISUI are implemented within the Hyperspectral sensor Electronics Unit (HELU). Science signals from detectors are converted into digital signals through high-speed analog-to-digital converters, and the digitized signals are processed inside the HELU.

Radiometric calibration and smile correction functions are realized by using RTAX2000S radiation-hardened FPGA (Field Programmable Gate Array) with JAXA (the Japan Aerospace Exploration Agency)-authorized four mega-byte burst static random access memories (BSRAMs).

Data binning in the spectral direction and lossless data compression are implemented within the HELU. The binning function is provided in order to transmit whole data, which consists of 186 spectral bands of VNIR and SWIR. Whole spectral bands are derived from every 2.5 nm for VNIR and 6.25 nm for SWIR, respectively, and those pixel data are processed through binning functions in a spectral direction over four pixels in VNIR and over two pixels in SWIR in order to produce a unity image pixel. The binning functions are implemented with smile correction functions, which result in flexible and small-size circuitry implementation.

## 3. A Novel Algorithm for Natural Image Compression

The image compression operation of high-speed sensor signal outputs for onboard satellite equipment should be processed in real-time, using relatively slow-speed radiation-hardened devices.

A good image compression ratio has been reported with a two-pass encoding algorithm [9]. Since real-time image compression with a speed of the camera rate of every 4.36 ms is required in order to process a large volume of output data from the HISUI sensor, a one-pass encoding algorithm with an improved compression ratio has been developed. The following section describes the algorithm.

### 3.1. Overview of Image Compression Algorithm

StarPixel^®^ is a newly developed compression method aimed at realizing a fast compression speed with a low-complexity algorithm [10]. It is developed for planetary exploration as well as Earth observation [11], and now it has widely been deployed for commercial appliances. Both the lossless method and lossy method are developed within the framework of StarPixel. Its main purpose is to achieve a fast processing speed while keeping almost the same compression ratio as JPEG-LS and JPEG2000 [12].

A fast compression algorithm and implementation are essential for realizing lightweight and small-size image compression units, because the number of encoder channels can be reduced. The high speed implementation enables multiplexing sensor signal outputs into reduced transmission channels, which results in reducing the mass of the signal processing unit. The image compression method should have coding efficiency, high compression speed, and low complexity in order to reduce the mass, size, power consumption, and fabrication cost of the onboard signal processing unit. Reducing the mass and size of payload modules incorporates agile pointing capability, and low power consumption provides a thermal stability preferable for optical observation satellites.

Conventional lossless image compression methods, such as JPEG-LS and JPEG2000, have high coding efficiency, whereas their insufficient compression speed and high complexity prevent the size, mass, and power consumption of compression units. The developed method is based on Fast, Efficient, Lossless Image Compression System (FELICS) [13], which is a simple hierarchical predictive coding method with resolution scaling. In order to improve FELICS’s performance of image decorrelation and entropy coding, we apply a two-dimensional interpolation prediction and adaptive Golomb-Rice coding.

The compression speed of StarPixel is almost 30 times faster than JPEG-LS or lossless JPEG2000, whereas the compressed data size using StarPixel is only 1% or 5% larger than that using JPEG-LS or lossless JPEG2000. Both software and hardware implementation has already been developed in order to encompass a wide range of needs from Earth observation to inter-planetary exploration. The first implementation was demonstrated on the S-520 sounding rocket, and now, onboard equipment for satellites is working in orbit for the Venus exploration satellite “AKATSUKI” [14] and the asteroid probe “HAYABUSA2” [15,16,17].

### 3.2. Image Modeling and Prediction

JPEG-LS and JPEG2000 are often used as efficient lossless compression methods among conventional image compression algorithms. JPEG-LS adopts prediction coding and achieves a high compression ratio using various mode selections with many branches. Discrete wavelet transform and coefficient bit plane arithmetic coding (Embedded Block Coding with Optimized Truncation (EBCOT)) are employed by JPEG2000 with a heavy processing load. Our novel algorithm is based on a prediction coding method without transformation, which leads to a light memory access load. Interpolation prediction is employed for the algorithm, and the compressor predicts and encodes each reduced-resolution image hierarchically, from the initial lowest resolution to the highest resolution of the original images. The compressor first encodes the initial resolution image, and then progressively encodes higher resolution (*i.e*., less subsampled) images by halving the subsampling interval until it reaches 1. A higher prediction precision is achieved than with JPEG-LS, which employs one-sided prediction. Symmetric geometry prediction is adopted for the algorithm, which eliminates complex mode selection as direction selections.

The algorithm aims at better lossless compression of natural images with lower complexity. Since the entropy of natural images is relatively high and the compression ratio is low, highly compressible bit-by-bit arithmetic coding is not necessary. Also, Huffman encoding is not suitable because it requires huge coding tables in order to process various color depth. Therefore, we adopt simple adaptive Golomb-Rice codes for the entropy coder. The implementation detail of our adaptive Golomb-Rice encoder is described in Section 3.3 and Section 3.4.

**Figure 1:**
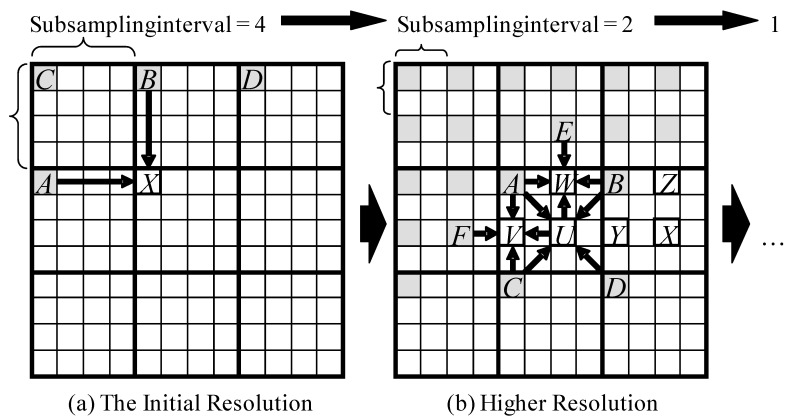
Hierarchical coding process and prediction operators.

Figure 1a shows the encoding process of the initial resolution image. For the initial resolution, some pixels of the original image are subsampled by a factor of 2*n* from left to right, then top to bottom. Each subsampled pixel is predicted as the average of two known pixels: the left-hand neighboring pixel A and the upper neighboring pixel B.

Figure 1b shows the encoding process of a higher resolution image. The pixels A–D are known because they were already encoded in a former resolution image, and E–F are known because they were already encoded at the current resolution. U–Z are the pixels to be coded. The compressor predicts each set of three unknown pixels around the known pixels in the following order:

P1: The pixel located at the midpoint of the four known pixels from the former resolution.
P2: The pixel located left of the preceding pixel P1.
P3: The pixel located above P1. Thus, the compressor encodes the pixels U, V, and W, and then X, Y, and Z.

Each unknown pixel is predicted as the average of four surrounding pixels. Pixel U is predicted as the average of A, B, C, and D, while V is predicted as the average of A, C, F, and U, and so on.

These predictors improve the decorrelation efficiency of FELICS from the three points of view:

(1) Coding efficiency: the prediction by four reference pixels performs better than FELICS’s prediction by two reference pixels, especially in the smooth area of natural images.
(2) Computational efficiency: no need to select two reference pixels like with FELICS. We can reduce conditional branches in the compression process.
(3) Memory access efficiency: by reusing the preceding pixels at the current resolution to predict the following pixels, each resolution scale can be coded by a single pass pixel scan, while FELICS needs two passes of pixel scan for each resolution scale.

### 3.3. Context Modeling

**Figure 2:**
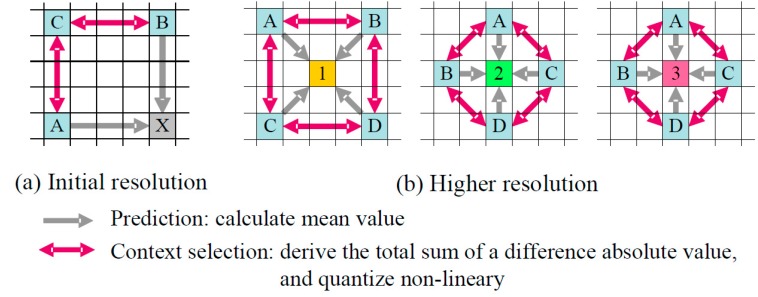
Decision process of coding context.

The coding context of each pixel is calculated as the quantized sum of the absolute differences between the known neighbor pixels. Figure 2 shows the detailed process.

For the initial resolution (Figure 2a), the coding context of X is to be the quantized sum of two absolute differences, between A and C, and between B and C.

For higher resolutions (Figure 2b), the coding context of 1 is the quantized sum of four absolute differences, between A and B, between C and D, between A and C, and between B and D.

The contexts of 2 and 3 are decided in the same way as 1, but are in another context group. These operations are geometrically symmetrical, and, therefore, single instruction-multiple data (SIMD) can be adopted in these process.

### 3.4. Entropy Coding

**Figure 3:**
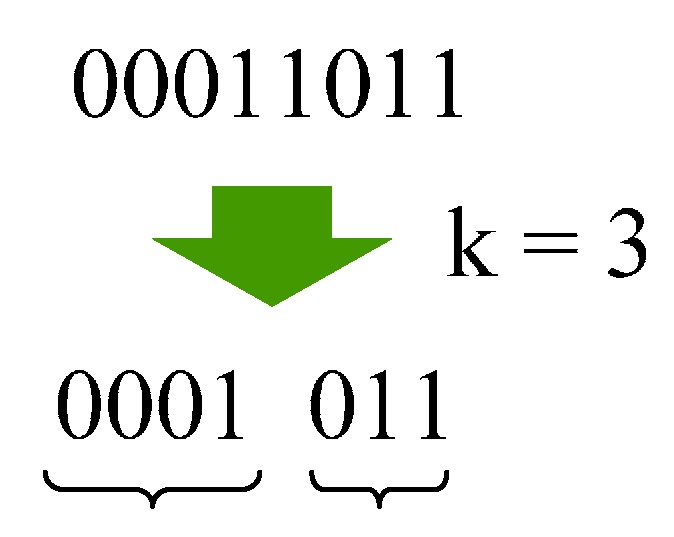
Golomb-Rice encoding.

The compressor encodes prediction errors with an adaptive Golomb-Rice coding method, which is similar to JPEG-LS. Golomb-Rice codes [18,19] for *N*-bit symbols with parameter *k* are defined as the combinations of unary representations of upper (*N* − *k*) bits and binary representations of lower *k* bits. Figure 3 shows the example of Golomb-Rice encoding in which parameter *k* equals 3. The coding sequence is as follows in this example,

(1) divide a symbol into (8 − *k*) bits and *k* bits,
(2) (8 − *k*) bits “00011_b_” equals 3, then 3 bits of “0” are generated,
(3) 1 bit “1” is added as a termination of the preceding “0”,
(4) lower 3 bits “001_b_” are added in the end.

The most efficient code is derived in the case that the *k*-value equals to actual effective digits. JPEG-LS performs an adaptive estimation of the optimal Golomb parameter *k*, calculating the average magnitude of recent prediction errors for each coding context. A bit shift operation and conditional branch occur during the mean value calculation, which require a high processing load.

The proposed method performs a faster estimation with some approximation. Unlike JPEG-LS, the compressor does not accumulate the magnitude of prediction errors, but instead accumulates the optimal parameters of recent prediction errors.

Since the values of accumulated parameters are much smaller than the accumulated magnitudes, the compressor can use a reasonably small lookup table in order to quickly decide the parameter from an accumulated value and an accumulation counter. This provides a significant performance increase (50% faster compression in most cases), with only a slight decline in estimation accuracy (0.5% decline in compression ratios).

## 4. Onboard Compensation and Binning

### 4.1. The Background of Onboard Compensation and Binning

A Complementary Metal Oxide Semiconductor (CMOS) sensor is used as a detector for VNIR, and a photo-voltaic mercury cadmium telluride (PV-MCT)-type linear array is selected for SWIR. The resolution of the detector supersedes the spatial frequency resolution of the optical structure, and the data rate of the output signal from the detectors also exceeds the downlink transmission capacity of the satellite. The binning function is provided in order to reduce the data volume over the spatial frequency, namely four pixels along the spatial frequency are added with an adequate weighting parameter for each pixel.

HELU makes the compensation of Photo Response Non-Uniformity (PRNU), non-linearity, and offset. In order to maintain the precision of the result of the binning process and compression efficiency, a radiometric calibration function is provided for correcting the sensitivity and linearity of each pixel.

A smile characteristics calibration function is also provided in order to compensate the distortion of the optical structure subsystem. The calibration is carried out at the same time as the binning operation without additional hardware circuitry.

### 4.2. Onboard Compensation Algorithm

Observation data derived by HISUI is transmitted through the binning process; therefore, the calibration for each element should be applied onboard before being processed with the binning function. The calibration process consists of sensitivity correction and smile correction, which are applied both on VNIR and SWIR data.

**Figure 4:**
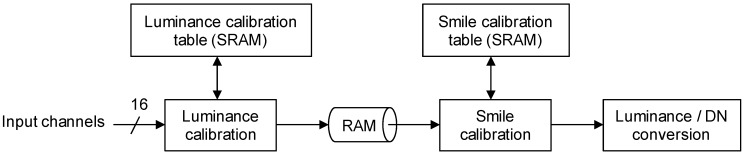
The block diagram of the compensation function.

**Figure 5:**
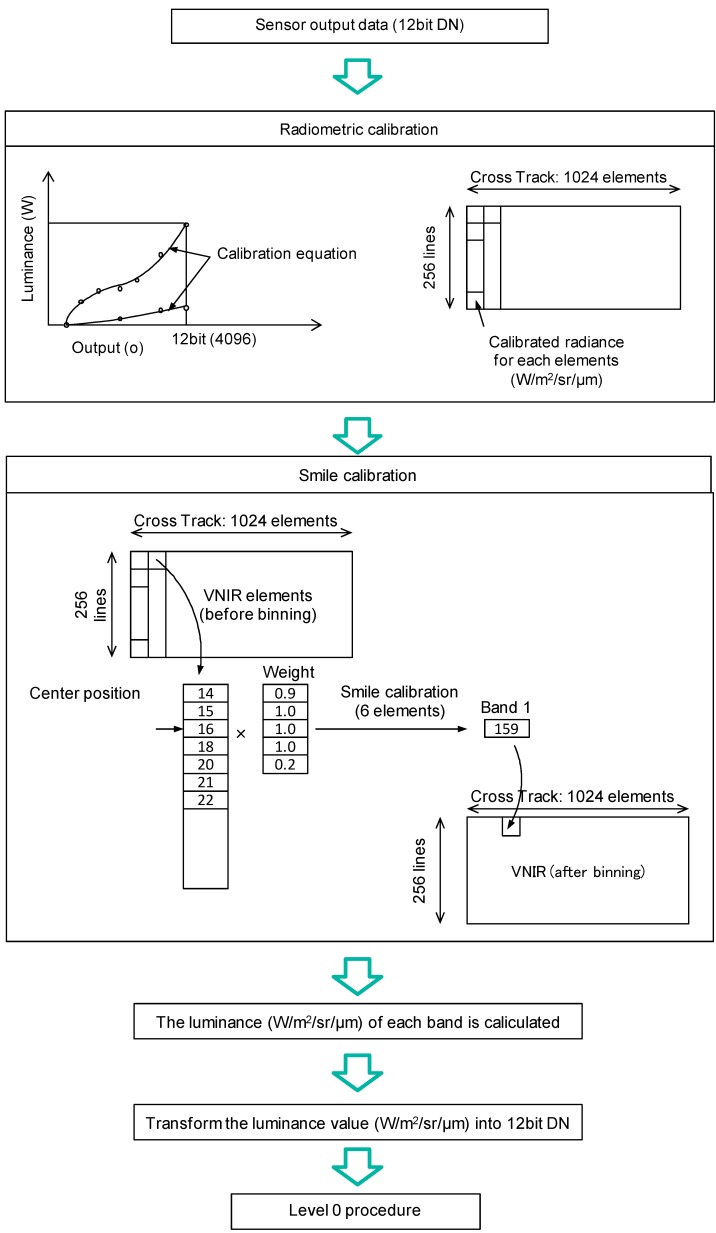
The radiometric calibration and the smile correction (VNIR case).

The onboard compensation function block diagram is shown in Figure 4. The function block is built into the control unit. Each element of the sensor has a 12-bit digital data output port. The sensitivity of each pixel is corrected using parameters stored in the table implemented with static random access memories (SRAMs). The corrected data are treated as the luminance of elements represented as 16-bit digital data. These corrected data are stored in ring buffers and relocated as required for post processing. The following process consists of smile correction and binning. The coefficients and tables for the smile correction and binning are read out from onboard SRAMs. The luminance of each element is compressed as a 12-bit digital number (DN) after the onboard calibration. The data are formatted as tiles and compressed by StarPixel^®^ lossless image compression. The compressed data are formatted in accordance with the Consultative Committee for Space Data Systems (CCSDS) recommendations [20]. The data flow of the onboard compensation is shown in Figure 5.

**Figure 6:**
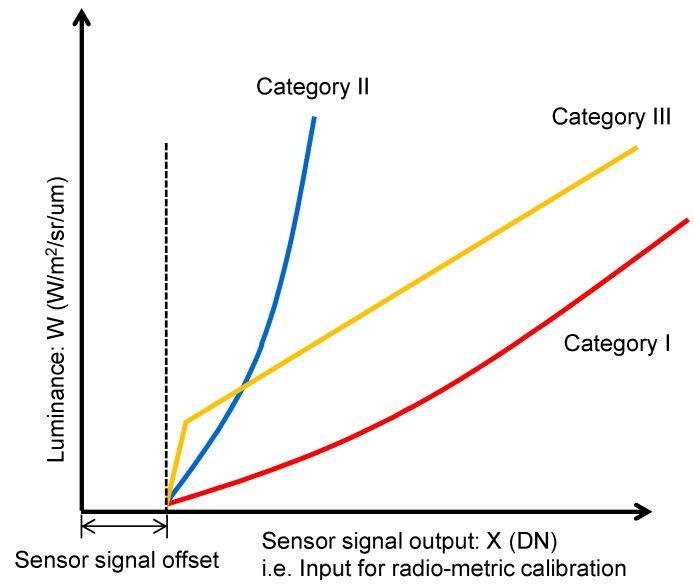
Sensitivity calibration scheme.

The processing scheme of sensitivity calibration is shown in Figure 6. The characteristics of all elements of CMOS detectors used on HISUI are evaluated through the production test in advance, and the characteristics are categorized into three sets: quadratic response with slight non-linearity, quadratic response with a wide range of the variation of non-linearity, and linear response with some offset. Almost 96% of the luminance characteristics of elements are represented by the quadratic equation shown in Equation (1), which is represented as a quadratic response with slight non-linearity and is distinguished as category I. The coefficients A1, B1, and C1 characterize the slight non-linearity and they are consolidated after statistical investigation of the luminance response of the pixels in category I. Another set is distinguished as category II, which is also represented by the quadratic equation, though its slope is steeper than category I and it has a wide range of the variation of non-linearity. Almost 3.8% of the pixels are grouped into category II. The coefficients A2, B2, and C2 characterize the wide range of variation of non-linearity, and they are also consolidated after statistical investigation of the luminance response of the pixels in category II. The third category, which is distinguished as category III, is represented by the combination of linear lines with two slopes. Almost 0.2% of the detector elements are categorized in this set. The coefficients A3, A4, B3, and C3 are derived after statistical investigation of the luminance response of the pixels in category III. The fixed value 50 shows the lower limit of offset, which is also derived through statistical investigation. The coefficients A1, A2, A3, A4, B1, B2, B3, C1, C2, and C3 are well suited to the CMOS sensors that we procured for HISUI, though they might depend on the conditions as a manufacturing lot and other conditions, so we have to evaluate coefficients for the regression of each category for every CMOS detector.

W = A1 × (X − C1)^2^ + B1 × (X − C1) (1)

W = A2 × (X − C2)^2^ + B2 × (X − C2) (2)

W = A3 × X + B3, W = A4 × (X − C3 + 50)
(3)

The coefficients for sensitivity calibration are represented in 32-bit digital data format and stored in the electrically erasable programmable read-only memories (EEPROMs) inside the control unit of HISUI.

## 5. Evaluation Result

### 5.1. Compression Performance

The lossless compression performances of several codecs have been evaluated. The evaluation result includes StarPixel-lossless, denoted here as HIREW, for various pixel formats, which are grayscale 8 bpp and 16 bpp, and RGB (Red-Green-Blue) color 8 bpp. All test images were quoted from the National Aeronautics and Space Administration/Jet Propulsion Laboratory - the California Institute of Technology (NASA/JPL-Caltech) website [21]. Some of the grayscale images were converted from the original color images.

We use an optimized implementation of each codec if possible [22,23,24], and execute it in a single thread. Note that the tested FELICS [13] is the non-progressive implementation, because no progressive implementations could be found publicly.

The configuration of the subsampling interval of the initial resolution of HIREW is set as 16, because the compression efficiency of HIREW saturates at the initial interval of 16.

**Figure 7:**
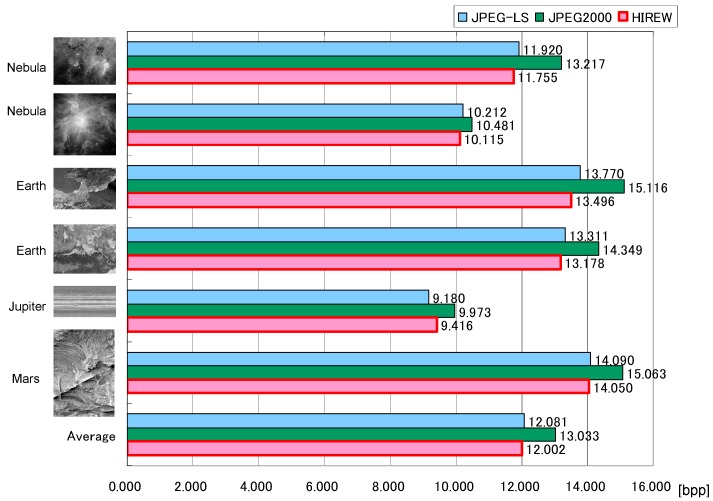
Comparison of lossless compression ratio for 16 bpp grayscale images (color images are pre-converted to grayscale 16 bpp).

**Figure 8:**
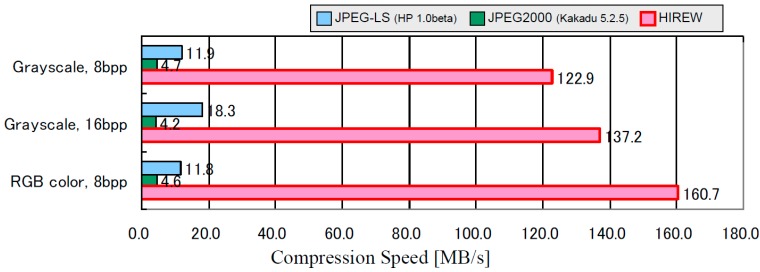
Comparison of lossless compression speed on Pentium 4, 3.6 GHz (Excluding file I/O).

Figure 7 shows the comparison of lossless compression ratios, and Figure 8 shows the comparison of compression speeds (MB/s) on a personal computer with a 3.6 GHz Pentium 4 processor. The performance comparison summary is derived as follows:

(1) Compression ratio: The compression ratio of HIREW is approximately the same as lossless JPEG2000 and JPEG-LS, with a slight advantage for HIREW for complex (*i.e*., less compressible) and high-precision grayscale images.
(2) Compression speed: The compression speed of HIREW is 26–35 times faster than lossless JPEG2000, and 7–14 times faster than JPEG-LS.

As for the coding efficiency of entropy encoding, Huffman encoding can manage arbitrary input signal distribution, and has the advantage of deriving the optimal compression ratio. On the other hand, a large memory capacity is required for coding tables in order to encode large bit-depth (16-bit) signals from optical sensor head modules. Satellite onboard equipment has the limitation of resources such as mass, power consumption, and size; nevertheless, onboard optical sensor heads often employ large bit-depth for the optical sensor head. As a result, we employ Golomb-Rice encoding instead of Huffman encoding due to the trade-off between coding efficiency and onboard resource limitation.

The compression speed is fast enough for the AKATSUKI Venus climate orbiter even though the compression unit is realized as a software module [14]. The result had an advantage for the inter-planetary mission. On the other hand, sufficient compression speed has been derived by FPGA implementation for HISUI in order to transmit large volume observation data in Low Earth Orbit (LEO) in the camera rate processing speed.

### 5.2. Compression Direction

**Figure 9:**
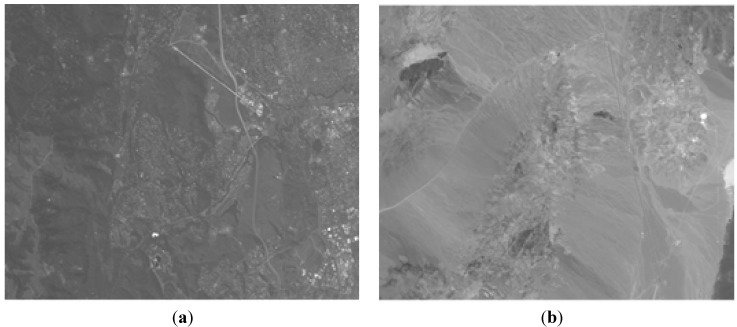
(**a**) Jasper, VNIR, the 31st band image; (**b**) Cuprite, SWIR, the 29th band image [25].

There are two candidates for the compression direction of hyperspectral data. One is spatial direction and the other is spectral direction. Hyperspectral image data derived from airborne sensors were used in order to evaluate the direction of compression. The images shown in Figure 9 are the examples of hyperspectral data provided by the courtesy of Japan Space Systems (Jspacesystems) [25]. One is the two-dimensional image of the 31st band of VNIR, which is the scene of Jasper, and the other is the 29th band image of SWIR, which is the scene of Cuprite.

The compression efficiency using the StarPixel software encoder was evaluated for two cases, which are denoted as cases A and B. Case A is compressed along the spatial direction. The compressed image data for case A consists of 614 × 512 two-dimensional elements. Case B is compressed along the spectral direction. The compressed image for case B consists of 614 elements multiplied by the number of bands, which is equivalent to the frame data composed of pixel data along with cross-track directions multiplied by the number of spectral bands.

The results of the simulation using the examples are shown in Table 2. No significant difference in the compression ratio is identified between cases A and B.

In contrast, the capacity of SRAMs for the compression process of the image data shows a remarkable difference in the implementation point of view. Case B requires the capacity equivalent to one frame, which corresponds to 186 bands multiplied by 1024 elements. Case A requires SRAM capacity, which corresponds to the case B capacity multiplied by frame numbers from 100 to 1000. Consequently, apparently a larger hardware resource is required for the implementation of case A than that of case B. The result led us to employ case B, which is the compression along the spectral direction.

**Table 2:** Compression ratio of case A and B using the examples.

Image	Case A	Case B
Cup, SWIR, 29 band:	6,841,790 (37.5%)	7,226,330 (39.6%)
Jasper, VNIR, 31 band:	11,905,838 (61.1%)	11,631,488 (59.7%)

### 5.3. Hardware Implementation Result

**Figure 10:**
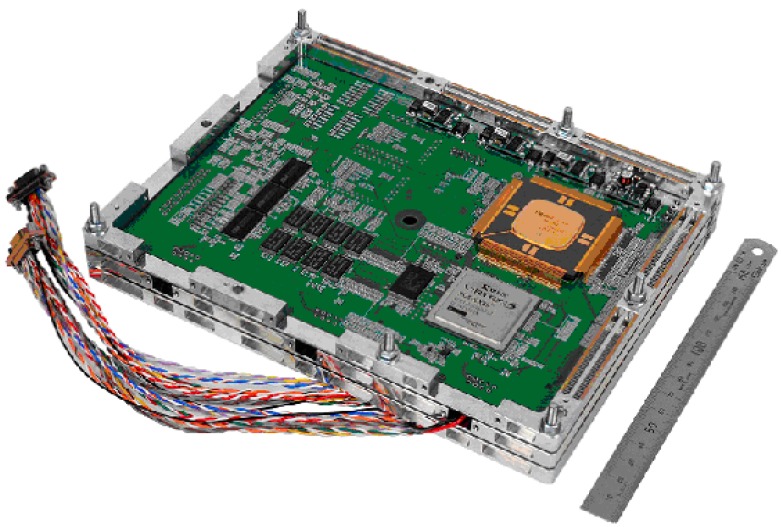
Hardware implementation of StarPixel-lossless encoder (an engineering model).

The engineering model for the verification of the resource efficiency using StarPixel has already been developed, as shown in Figure 10. StarPixel-lossless has been implemented on Xilinx FPGA, which corresponds to the same capacity as ACTEL RTAX2000S radiation-hardened FPGA used for space applications. JAXA-authorized radiation-hardened 64-bit micro-processor HR5000S has been used for the control of the compressor FPGA, as shown in Figure 10 for the engineering model. The 96 MHz FPGA implementation achieved 180 k gate counts (10 times smaller than lossless JPEG2000 on an ASIC), and 76 M pel/s at throughput speeds (10 times faster than lossless JPEG2000 on an ASIC).

The engineering model has formatting functions for transmitting sensor data. As a result, the possibility of embedding compressor circuitry into the front-end of the electronics unit has been verified using radiation-hardened devices for space use.

**Figure 11:**
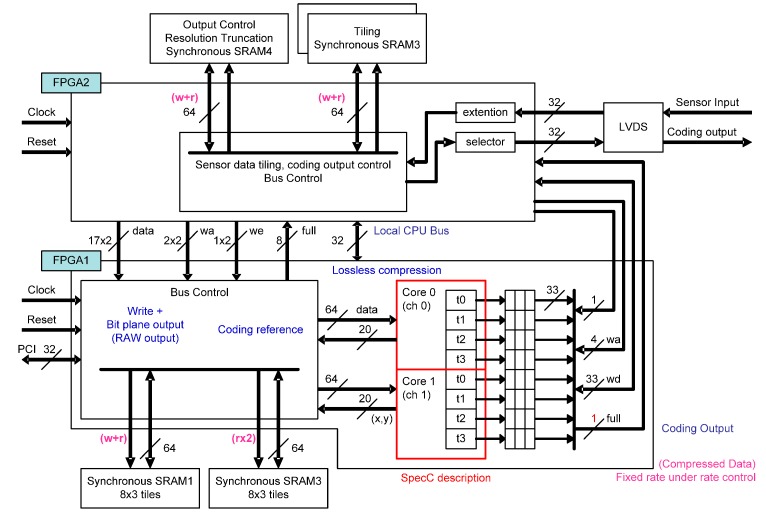
The block diagram of hardware implementation of the StarPixel-lossless encoder (an engineering model).

The block diagram of the engineering model is shown in Figure 11. The hardware implementation aims at processing image pixels in a so-called camera rate. Therefore, the amount of encoded data should be transmitted within the capacity of downlink transmission to ground stations, and the transmission rate control capability is required. In order to keep image quality as well as an efficient transmission rate, pixel values are divided into a higher-layer bit-plane and lower-layer bit-plane. The pixel values in the higher layer are compressed using StarPixel encoding, and the values in the lower layer remain uncompressed. Compressed data in the higher layer has a high priority for downlink transmission, and each bit-plane is transmitted in accordance with its priority. The raw pixel values are transmitted without compression, and once the reserved transmission capacity has been occupied during transmission, the pixel data with lower priority are truncated. The truncation in the higher layer corresponds to the resolution truncation, because complete resolution in the higher layer is kept provided that all data in the higher layer are transmitted for decoding. The truncation in the lower layer is simple bit-plane truncation, so the resolution and dynamic range depends on the remaining transmission capacity of the downlink channels. The effective bit-depth is often smaller than hardware implementation; for example, 12-bits are used from 16-bit depth data. Therefore, the layering method and truncation scheme work well enough for natural image sensor applications.

We used behavioral synthesis tools [26,27] for implementing the compression core circuit directly from C-language implementation for accelerating implementation. The design framework enables us to provide parallel implementation within a limited development time frame. Since the design is transformed into real hardware implementation through behavioral synthesis tools, we evaluated the hardware implementation performance using high-level language implementation. The performance expected using space-qualified ASICs with the JAXA authorization process is 50 MHz to 60 MHz for the operation frequency of a source clock. The clock rate is not enough for the performance described in the preceding section. Consequently, we implemented four parallel streams into one core in order to derive the required compression speed. The parallel implementation scheme is also shown in Figure 11. The parallel implementation has been achieved with a straightforward process using behavioral synthesis tools.

The flight model of the electronics unit of HISUI is under development, and the processing speed is evaluated using the engineering model. The camera rate image compression performance is verified, which works at 25 MHz on the radiation-hardened flight-qualified RTAX2000S FPGA. The data rate shown in Table 1 is not degraded, and the onboard signal processing speed is maintained. As a result, the compression speed of StarPixel with a small footprint is qualified, and this eliminates the necessity of developing ASICs.

### 5.4. The Performance of the Onboard Compensation Algorithm

We have almost a flat luminance response with the above compensation at this time, but the compensation equations and coefficients might vary over CMOS detectors. Despite the variation, the three types of regression functions are proper enough both for the correction of linearity of flight-level CMOS detectors and hardware implementations.

The compensation must be carried out with the camera rate speed in spite of using radiation-hardened devices for space use whose operating speed is relatively slower than that of commercial devices. Therefore, the simple implementation of the linear equation and quadratic equation is desirable as long as they are good enough for deriving a modest linear response. Higher order regression functions might have a better compensation performance, whereas more than one pole requires a longer evaluation and test duration. We avoided the risk of uncertainty on the development schedule this time, and the systematic characterization of regression functions are a future issue.

## 6. Conclusions

The developed lossless encoder achieved a significantly faster compression speed than existing compression methods by applying hierarchical interpolating prediction and simplified adaptive Golomb-Rice encoding. The compression ratio is equivalent to JPEG2000 lossless coding.

Due to the small-footprint circuitry based on the algorithm, the compression function and the compensation function are embedded into the front-end circuitry of the formatter function within the electronics unit of the hyperspectral sensor. The result leads to compact hyperspectral sensor realization without providing an additional independent unit for lossless compression encoding.
